# The papain-like cysteine protease CEP1 is involved in programmed cell death and secondary wall thickening during xylem development in Arabidopsis

**DOI:** 10.1093/jxb/ery356

**Published:** 2018-10-30

**Authors:** Jingyi Han, Hui Li, Bin Yin, Yongzhuo Zhang, Yadi Liu, Ziyi Cheng, Di Liu, Hai Lu

**Affiliations:** 1 College of Biological Sciences and Biotechnology, Beijing Forestry University, Beijing, China; 2 Beijing Advanced Innovation Center for Tree Breeding by Molecular Design, Beijing Forestry University, Beijing, China

**Keywords:** Cysteine protease, programmed cell death, secondary wall, xylem development

## Abstract

Both tracheary elements and fiber cells undergo programmed cell death (PCD) during xylem development. In this study we investigated the role of papain-like cysteine protease CEP1 in PCD in the xylem of Arabidopsis. CEP1 was located in the cell wall of xylem cells, and *CEP1* expression levels in inflorescence stems increased during stem maturation. *cep1* mutant plants exhibited delayed stem growth and reduced xylem cell number compared to wild-type plants. Transmission electron microscopy demonstrated that organelle degradation was delayed during PCD, and thicker secondary walls were present in fiber cells and tracheary elements of the *cep1* mutant. Transcriptional analyses of the maturation stage of the inflorescence stem revealed that genes involved in the biosynthesis of secondary wall components, including cellulose, hemicellulose, and lignin, as well as wood-associated transcriptional factors, were up-regulated in the *cep1* mutant. These results suggest that CEP1 is directly involved in the clearing of cellular content during PCD and regulates secondary wall thickening during xylem development.

## Teaser

The papain-like cysteine protease CEP1 is involved in clearing cellular contents during programmed cell death and regulating secondary wall thickening during xylem development.

## Introduction

Secondary growth is an important biological process in woody plants. The wood that it produces provides raw material for construction, fuel, and paper manufacturing. During secondary growth, the cambium contains a pool of pluripotent stem cells that continuously produce xylem and phloem, resulting in thickening of the stem and roots and thereby increasing plant biomass (Miyashima *et al.*, 2013). Secondary growth is also observed in herbaceous Arabidopsis, which provides an excellent model system for this process (Nieminen *et al.*, 2004). Secondary xylem development involves a cascade of processes including cell wall expansion, secondary wall deposition, lignification, and plant cell death (Turner *et al.*, 2007; Bollhöner *et al.*, 2012). Several transcription factors have been found to regulate secondary xylem development (Demura and Fukuda, 2007; Zhong *et al.*, 2008), and plant hormones such as auxin, cytokinin, brassinosteroids, and ethylene are also involved in the process (Milhinhos and Miguel, 2013).

Both tracheary elements (TEs) and fiber cells undergo programmed cell death (PCD) during xylem development. PCD during TE differentiation has been extensively studied in the Arabidopsis model system, in poplar, and in *Zinnia elegans* cultured *in vitro*. During TE differentiation, collapse of the vacuole is considered the initial moment of cell death during PCD. The release of hydrolytic enzymes stored in the vacuole leads to the rapid degradation of organelles, nuclear DNA, and part of the cell wall (Fukuda, 1997; Kuriyama, 1999; Obara *et al.*, 2001; Escamez and Tuominen, 2014). Several cysteine proteases have been implicated in the regulation of xylem PCD during TE differentiation. The PCD-specific hydrolytic cysteine protease ZCP4 accumulates in the vacuoles of developing TEs in *Z*. *elegans* and plays a role in the degradation of cell contents following vacuolar collapse (Ye *et al.*, 2002). A peptide aldehyde inhibitor of papain-type peptidases blocks autolysis of TEs in *Z*. *elegans* cultures (Woffenden *et al.*, 1998). A protease with caspase-3-like activity is associated with PCD during secondary xylem development in poplar (Han *et al.*, 2012). Two xylem-specific papain-like cysteine peptidases, *XYLEM CYSTEINE PROTEASE1* (*XCP1*) and *XCP2*, have been shown to localize to vacuoles and act as effectors of autolysis during TE differentiation in Arabidopsis (Funk *et al.*, 2002; Avci *et al.*, 2008). Arabidopsis metacaspase 9 (AtMC9) is a xylem-specific metacaspase involved in regulating TE autolysis, and its loss of function results in defects in this process (Bollhöner *et al.*, 2012). Although several proteases have been characterized in PCD during xylem development, the full extent of the involvement of cysteine proteases remains unknown.

It has been previously determined that CEP1, a papain-like cysteine protease characterized by a C-terminal KDEL endoplasmic reticulum retention signal, is expressed in Arabidopsis roots, flowers, and stems, and is involved in tapetal PCD and pollen development (Zhang *et al.*, 2014). In this study, we found that mutations in *CEP1* delayed plant growth and altered xylem cell number. To determine whether *CEP1* is also involved in PCD during xylem development in Arabidopsis, we compared the structure and global transcriptional expression patterns of the inflorescence stem of *cep1* mutants with wild-type plants. Transmission electron microscopy revealed that the mutant plants displayed delayed organelle degradation during PCD and had thicker secondary walls in fiber cells and TEs. Many genes regulating the biosynthesis of secondary wall components were up-regulated in the *cep1* mutant. These results indicate that CEP1 is important in degrading the cellular content during PCD of secondary xylem growth and that it affects secondary wall deposition.

## Materials and methods

### Plant material and growth conditions

Seeds of the *Arabidopsis thaliana* T-DNA insertion mutants *cep1* (SALK_013036) and *cep1-2* (SALK_137016) were obtained from the Arabidopsis Biological Resource Center (https://abrc.osu.edu). The mutants were confirmed as homozygous by PCR using the primers BP, ATTTTGCCGATTTCGGAAC; LP1, TAGCAACAGCGAAAGGTAAGC, and RP2, AAGCTGTTGCTAATCAGCCTG for *cep1*, and BP, ATTTTGCC GATTTCGGAAC; LP3, CAAAATCTTAGTTTCGACGATGG; and RP4, GGCTCCATTCTTTCTCCAATC for *cep1-2*. Transgenic Arabidopsis seeds overexpressing CEP1 or the ProCEP1-GFP (green fluorescent protein) fusion construct were generated in our laboratory as described previously (Zhang *et al.*, 2014). Columbia-0 was used as the wild-type control. All plants were grown in a greenhouse at 23 °C (16 h light, 8 h dark, 120 µmol photons m^−2^ s^−1^).

### Real-time PCR analyses

Total RNA was isolated from the main stem at different plant developmental stages using an EASYspin Plant RNA Kit RN09 (Aidlab, China) according to the manufacturer’s instructions. First-strand cDNA synthesis was performed using a FastQuant Reverse Transcription Kit (Tiangen Biotech, Beijing, China). The mRNA level was normalized using the Arabidopsis *actin* gene (forward primer 5′- CGTATGAGCAAGGAGATCAC-3′ and reverse primer 5′- CACATCTGTTGGAAGGTGCT-3′). To detect *CEP1* expression in the real-time PCR assay the forward primer was 5′-CTATTGATGCTGGAGGCTCAGACT-3’ and the reverse primer was 5′-GAATCCCTCTCTGCATTCTTATGT-3′. SuperReal PreMix Plus (SYBR green; Tiangen Biotech) was used for the real-time PCR reaction, which was performed as follows: initial denaturation step for 15 min at 94 °C, followed by 40 cycles depending on the template with a denaturation step (30 s at 94 °C), an annealing step (20 s at 56 °C), and an extension step (20 s at 72 °C). A solubility curve was then calculated for the reactions, which concluded with a step of 30 s at 95 °C and a step of 30 s at 65 °C, followed by a step of slowly increasing temperature to 95 °C in increments of 0.5 °C s^–1^. Three plants were tested and each sample was analysed three times. Data were analysed using iQ5 software (Bio-Rad Laboratories, Hercules, CA, USA), and differences in gene expression were calculated using the 2^−ΔΔ*C*T^ method (Livak and Schmittgen, 2001).

### Stem histochemistry

For histological analyses, we collected the first basal-end node of the main inflorescence stem, immediately above the uppermost rosette leaf, at different developmental stages from the wild-type, the *cep1* T-DNA insertion mutant lines, and the *CEP1*-overexpressing transgenic lines. The basal nodes of inflorescence stems from early to late developmental stages were selected to produce paraffin sections. All samples were fixed in formaldehyde/acetic acid/alcohol fixation buffer (FAA; 70% ethanol:formaldehyde:acetic acid = 90:5:5) for 24 h at 4 °C and stored in 70% ethanol. The stem nodes were gradually dehydrated (70%, 80%, 95%, and 100% ethanol, 1 h each step) and 0.1% safranine O in ethanol was added to pre-stain the xylem. The stem nodes were then embedded in paraffin (Sigma-Aldrich) according to the manufacturer’s instructions. Transverse serial sections (8 μm thick) were prepared using a Leica RM2016 microtome, dewaxed in dimethylbenzene, mounted in neutral balsam after drying, placed on a cover glass, and observed under a Leica DCF500 microscope. Micrographs of sections from 9–12 specimens were examined. Statistical differences were determined using Student’s *t*-test.

### CEP1 reporter line and confocal laser scanning microscopy

The reporter line ProCEP1-GFP in the Col-0 background has been described previously by Zhang *et al.* (2014). Stems of transgenic Arabidopsis plants were examined using a Leica DMI6000 CS confocal laser-scanning microscope. GFP was excited with an argon laser at a wavelength of 488 nm, and emission was detected at 500 nm and 530 nm.

### Transmission electron microscopy

When the Arabidopsis plants had grown to stage 4 and the inflorescence stem had stopped flowering and elongating, the first basal stem nodes were collected for TEM analyses. The nodes were pre-fixed in 3% (w/v) paraformaldehyde and 0.25% glutaraldehyde in 0.2 N sodium phosphate buffer (pH 7.0), then fixed in 2% osmic acid in phosphate-buffered saline for 3 h. After rinsing with phosphate buffer, samples were gradually dehydrated in ethanol (30%, 50%, 70%, 80%, 90%, and 100%, 1 h each step). The ethanol was then gradually replaced by propylene oxide (25%, 50%, 75%, and 100%, 10 min each step) and samples were embedded in Spurr resin (SPI-Chem^TM^ Low Viscosity Spurr Kits, SPI Supplies, West Chester, PA, USA) according to the manufacturer’s instructions. Ultrathin sections (70 nm) were obtained using a Leica UC6 ultramicrotome and double-stained with 2% (w/v) uranyl acetate and 2.6% (w/v) lead citrate aqueous solution. Observations and image capture were performed with an H-7650 transmission electron microscope at 80 kV and a 832 charge-coupled device camera (Hitachi). Cell wall thicknesses were determined as the mean value of four measurements taken perpendicularly across the wall in each cell. Cell size and cell wall thickness were measure using the ImageJ software (https://imagej.nih.gov/ij).

### Transcriptome analyses

When the Arabidopsis plants had grown to stage 4 and stopped flowering, the stems were collected for transcriptional analyses. Total RNA was isolated using an EASYspin Plant RNA Kit RN09 (Aidlab) according to the manufacturer’s instructions. Sequencing libraries were generated using a NEBNext Ultra^TM^ RNA Library Prep Kit (New England Biolabs, Ipswich, MA, USA) according to the manufacturer’s instructions. The samples were sequenced using a HiSeq 2000 Genome Analyzer (Illumina, Inc., San Diego, CA, USA). High-quality clean reads were selected and mapped to the Arabidopsis reference genome and reference genes using TopHat v2.0.12 (Trapnell *et al.*, 2009). Gene expression levels were calculated as reads per kb per million reads (Trapnell *et al.*, 2012). The data were deposited in the NCBI Gene Expression Omnibus database (https://www.ncbi.nlm.nih.gov/geo/query/acc.cgi?acc=GSE102694) under accession number GSE102694.

Differential expression analyses between the *cep1* mutant and wild-type plants were performed using the DEGseq R package based on normalized read counts (Wang *et al.*, 2010). *P*-values were adjusted using the Benjamini and Hochberg approach for controlling the false discovery rate (FDR). A corrected *P*-value of <0.05 and a log_2_ ratio ≥1 or ≤–1 were set as the thresholds for significant differential expression. Cluster analyses of the gene expression patterns were performed based on the *K*-means method using Cluster 3.0 (Sturn *et al.*, 2002). Gene ontology (GO) enrichment analyses of the differentially expressed genes (DEGs) were performed using the GOseq R package (Young *et al.*, 2010). GO terms with a corrected *P*-value of <0.05 were considered significantly enriched among DEGs. The KOBAS 3.0 software (http://kobas.cbi.pku.edu.cn/) was used to test for the significance of enrichment of DEGs in the Kyoto Encyclopedia of Genes and Genomes (KEGG) pathways.

### Accession numbers

Sequence data from this article can be found in the Arabidopsis Genome Initiative under the accession number At5G50260 (*CEP1*).

## Results

### *CEP1* expression patterns in Arabidopsis stems and the *cep1* mutant phenotype

**Fig. 1.**
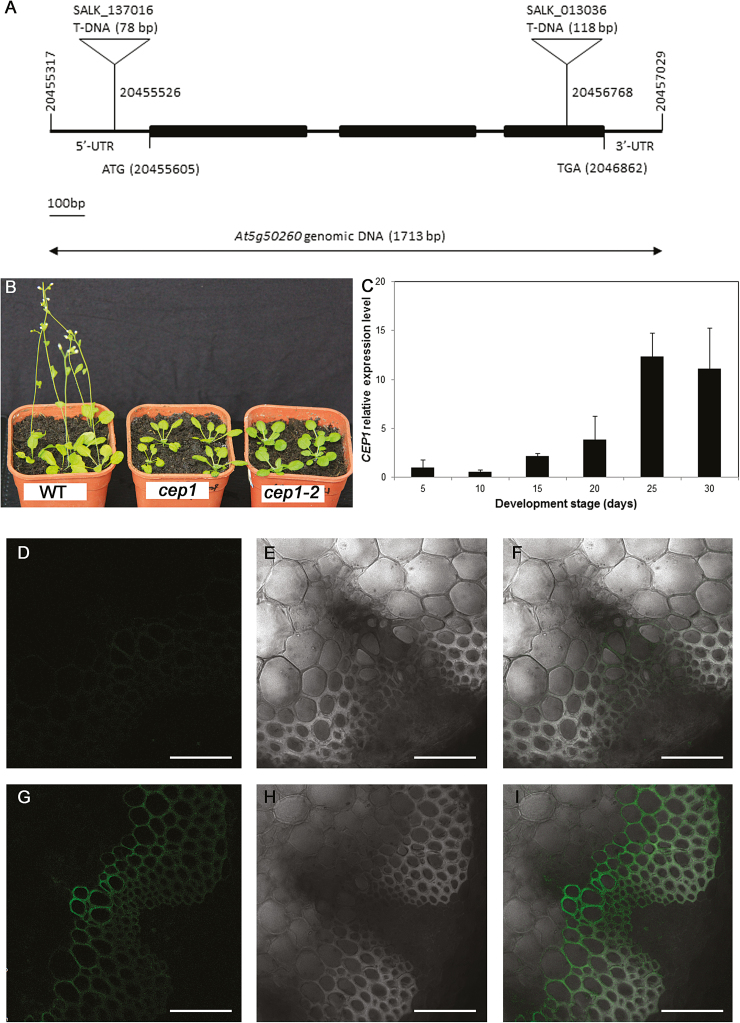
The phenotype of Arabidopsis *cep1* mutant plants and CEP1 expression patterns. (A) Structure of the T-DNA insertion sites in *cep1* and *cep1-2*. Exons are represented by the black bars. (B) Plants of the wild-type (WT), *cep1*, and *cep1-2* at 4 weeks old. (C) *CEP1* gene expression in the inflorescence stems of the wild-type at different time points after plant bolting (day 0). The relative expression level of *CEP1* at each time point was compared to that of at 5 d after bolting. (D–I) Green fluorescent protein (GFP) signals were detected in the vascular tissues of ProCEP1-GFP transgenic Arabidopsis plants (G–I) but not in those of wild-type plants (D–F). (D, G) GFP channel, (E, H) bright channel, and (F, I) overlay of GFP and bright channels. Scale bars are 50 μm.

We examined the phenotypes of the mutant lines *cep1* and *cep1-2*, in which T-DNAs are inserted into the third exon and the 5′ untranslated region of *CEP1*, respectively (Fig. 1A), and found that both displayed significantly decreased *CEP1* expression levels. Compared to wild-type (WT) plants, the germination and flowering times of the mutants occurred 1 week later (Fig. 1B). However, no differences were observed in plant height and stem thickness between the mutants and the WT by the final stage of plant growth.

A previous study reported that *CEP1* is highly expressed in flowers, where it plays an important role in tapetal PCD and regulates pollen development, and it has also been detected in vascular tissues, including the roots and stem, but not in the leaves (Zhang *et al.*, 2014). To investigate whether *CEP1* was involved in regulating stem development, we analysed its expression in the inflorescence stem using quantitative real-time PCR from the initiation of flowering until the stem stopped elongating (Fig. 1C). Expression increased during inflorescence stem development and reached a maximal level when the stem stopped elongating at 25–30 d after bolting, when TEs are formed via PCD (Chaffey *et al.*, 2002). To investigate the location of CEP1 within the stem, the ProCEP1-GFP reporter line was used and the GFP signal was detectable in the wall of xylem cells, but not in the other cells of the vascular tissue (Fig. 1D–I). Accumulation of *CEP1* mRNA predominantly occurred during the late stage of stem development and ProCEP1-GFP was detectable in xylem cells, which suggested that CEP1 could be involved in xylogenesis during stem development.

### Mutation of *CEP1* alters stem growth and cell number during xylem development

**Fig. 2.**
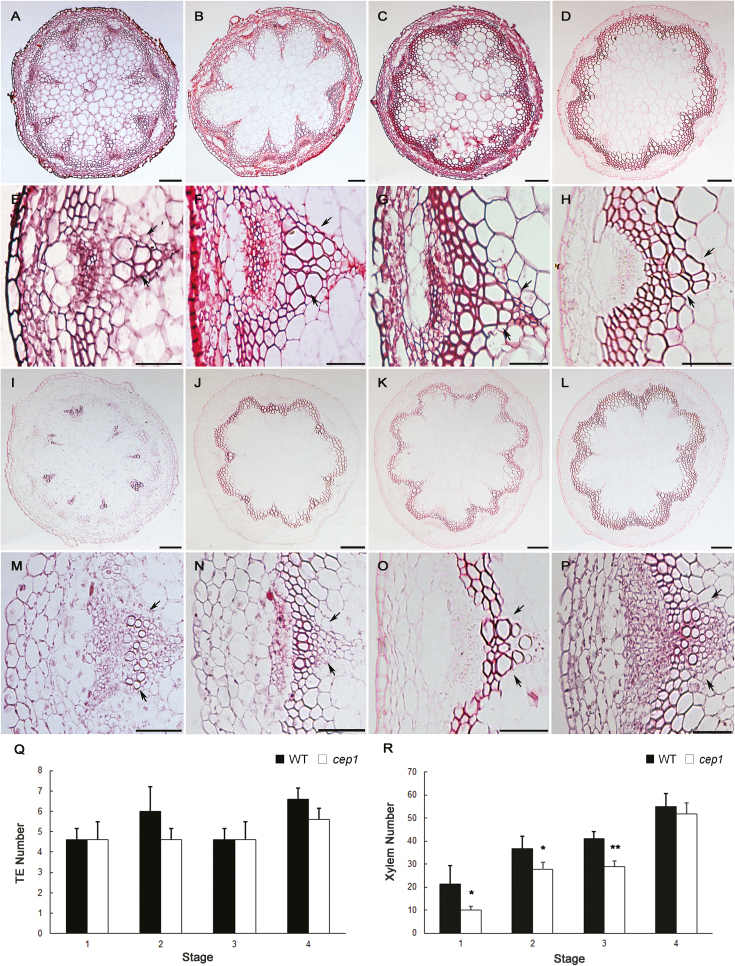
Histological analysis of the basal nodes of the stem in wild-type and *cep1* mutant plants at various developmental stages. (A–D) Cross-sections of the wild-type and (I–L) the *cep1* mutant. Scale bars are 100 μm. (E–H) High magnification images of vascular bundles in the wild-type and (M–P) the *cep1* mutant. Scale bars are 50 μm. The images are at stage 1 (A, E, I, M), stage 2 (B, F, J, N), stage 3 (C, G, K, O), and stage 4 (D, H, L, P). The arrows show the area of the xylem cells. (Q) The number of tracheary elements (TEs) in wild-type (WT) and *cep1* plants. (R) The total number of xylem cells in wild-type and *cep1* plants. Data are means (±SD) of three replicates. Significant differences were determined using Student’s *t*-test: *P<0.05; **P<0.01.

To investigate whether the morphology of the inflorescence stem was affected by mutations in *CEP1*, the basal nodes of stems at four different developmental stages were selected and paraffin sections were stained with safranine in order to examine the anatomy of the xylem (Fig. 2A–P). Given that the growth of *cep1* mutant plants was delayed by 1 week relative to the WT, we collected material based on developmental stage as described previously (Altamura *et al.*, 2001), with some modifications. Following anthesis of the first flower, stem development can be divided into four different stages based on morphological landmarks (Altamura *et al.*, 2001). It took 5 d and 12 d after bolting for WT and *cep1* plants to reach stage 1, respectively, and it took 30 d and 38 d after bolting for WT and *cep1* mutants to reach stage 4, respectively. Hence, stems from the *cep1* mutant were collected 1 week later than those of the WT plants at each stage. At stage 1, the first flower was observed. At stage 2, the first silique differentiated on the inflorescence stem. At stage 3, about 10 green siliques were observed on the inflorescence stem and apical floral buds were still present. At stage 4, when the inflorescence stem stopped flowering and elongating, most siliques were dehiscent and no apical buds were present. The heights of inflorescence stems from stages 1–4 were approximately 2, 12, 22, and 35 cm in both the WT and the *cep1* plants. Histological analyses revealed that both the *cep1* and *cep1-2* mutants and the WT plants differed in the shape and number of xylem cells at the different developmental stages (Fig. 2, Supplementary Fig. S1 at *JXB* online). The xylem cells in the *cep1* mutants were irregularly shaped compared to the WT. In particular, at stage 4 the TEs in the WT exhibited a polygon shape, while those in the *cep1* mutant appeared round. No significant differences were observed in the number of TEs (Fig. 2Q) but the total number of xylem cells in the *cep1* mutant, including TEs and xylary fibers in the vascular bundles, decreased significantly compared to the WT for stages 1–3; however, no difference was observed at stage 4 when the stem stopped elongating (Fig. 2R). This suggested that the mutation of *CEP1* only delayed xylem cell proliferation during early inflorescence development but did not alter the final cell number. This may explain why there were no final differences in height and stem thickness between the *cep1* mutant and the WT plants.

### Degradation of cellular contents is delayed in fiber cells and TEs of *cep1* mutant plants

**Fig. 3.**
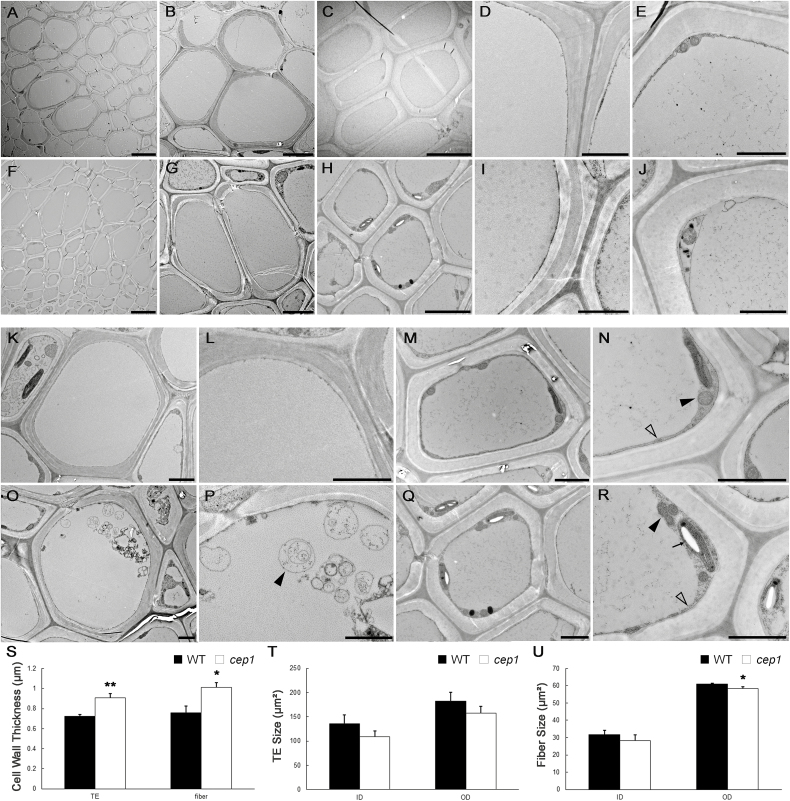
Transmission electron microscopy (TEM) of fiber cells and tracheary elements (TEs) of wild-type and *cep1* mutant plants. Fiber cells and TEs from the basal nodes of stage 4 plants were sectioned and examined. (A–E) Wild-type plants: (A) xylem cells, and (B) high-magnification images of TEs and (C) fiber cells, and the cell walls of (D) TEs and (E) fiber cells. (F–J) *cep1* mutant plants: (F) xylem cells, and high-magnification images of (G) TEs and (H) fiber cells, and the cell walls of (I) TEs and (J) fiber cells. (K–N) Wild-type plants: a TE lacking cellular content (K) and a fiber cell with cell contents, high-magnification image of a TE without cellular content (L), and mitochondria (solid arrowhead) and tonoplasts (open arrowhead) in a fiber cell (N). (O–R) *cep1* mutant plants: a TE with vesicles (O) and a fiber cell (Q) with cellular contents, vesicles (arrowhead) in a TE (P), and mitochondria (solid arrowhead), tonoplasts (open arrowhead), and plastids with amyloplasts (arrow) in a fiber cell (R). Scale bars: (A, F) 5 μm, (B, C, G, H) 5 μm, and (D, E, I–R) 2 μm. (S) Cell wall thickness of TEs and fiber cells in wild-type (WT) and *cep1* plants. Sizes of TEs (T) and fiber cells (U) in wild-type and *cep1* plants. Data are means (±SD) of three replicates. Significant differences were determined using Student’s *t*-test: *P<0.05; **P<0.01.

To investigate the differences in xylem cell morphology between *cep1* mutant and WT plants, TEM was performed (Fig. 3). The results of our expression pattern analyses suggested that CEP1 might be involved in PCD during stem maturation. To avoid the differences in development between the *cep1* mutant and WT plants, we focused on stage 4, when the inflorescence stem had stopped elongating. By measuring the internal and external area of xylem cells using TEM, we confirmed that the xylem cells were slightly smaller in the *cep1* mutant, particularly the fiber cells (Fig. 3A, F, T, U). Secondary wall thickness also appeared to be altered in fiber cells and TEs (Fig. 3D–E, I–J), with increases of 32.9% and 26.4%, respectively, in the *cep1* mutant relative to the WT (Fig. 3S).

CEP1 is a KDEL-tailed cysteine protease involved in PCD during tapetal development, and *cep1* mutants have been shown to abort tapetal PCD (Zhang *et al.*, 2014). To investigate whether CEP1 was also involved in cell death during xylogenesis, we examined the cellular degradation of *cep1* mutant during xylem development using TEM. TEs and fiber cells are characterized by having three layers of of relatively thick secondary walls, which can be used to distinguish them from parenchyma cells (Ye *et al.*, 2002). The cellular contents clearly disappeared in mature TEs of WT plants (Fig. 3K, L), whereas vesicles were still occasionally observed in the TEs of the *cep1* mutant (Fig. 3O, P). During the formation of fiber cells in WT plants, organelles gradually degrade before loss of integrity of the tonoplast occurs (Courtois-Moreau *et al.*, 2009). Hence, it was difficult to observe many organelles in mature fiber cells with thicker secondary walls. Only a few mitochondria, plastids, and non-degraded nuclei were observed in the cytoplasm of fiber cells when the tonoplast was intact in WT plants (Fig. 3M, N). By contrast, except for mitochondria, many plastids that had accumulated amyloplasts were found in mature fiber cells of the *cep1* mutant, which were rarely observed in the fiber cells of the WT (Fig. 3Q, R). This observation suggested that PCD was delayed in fiber cells and TEs in the *cep1* mutant.

### Overexpression of *CEP1* slightly affects PCD but does not alter xylem structure

**Fig. 4.**
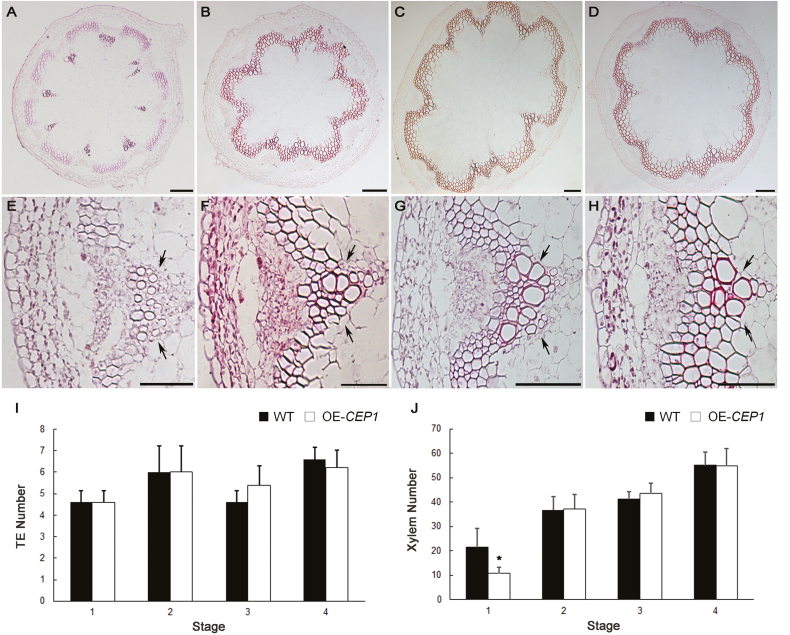
Histological analysis of the basal nodes of the stem of *CEP1*-overexpressing (*CEP1*-OE) plants at various developmental stages. (A–D) Cross-sections of the stem; scale bars are 100 μm. (E–H) High-magnification images of vascular bundles; scale bars are 50 μm. The images are at stage 1 (A, E), stage 2 (B, F), stage 3 (C, G), and stage 4 (D, H). The numbers of (I) tracheary elements (TEs) and (J) xylem cells for wild-type (WT) and *CEP1*-OE plants. Data are means (±SD) of three replicates. Significant differences were determined using Student’s *t*-test: *P<0.05.

**Fig. 5.**
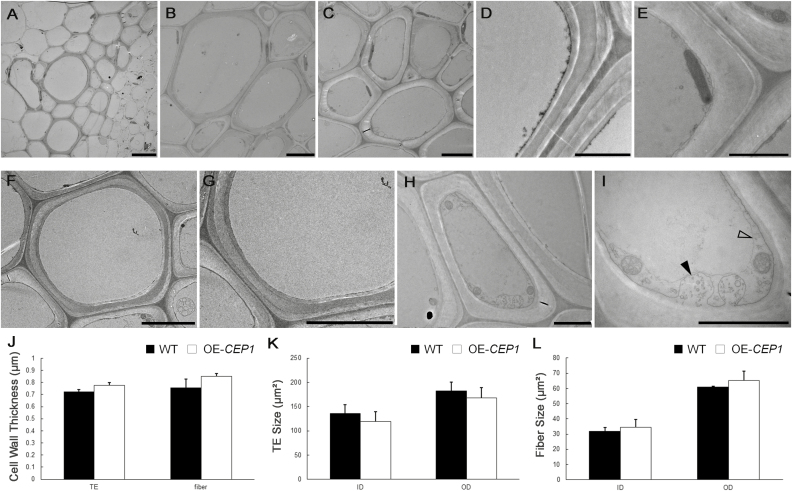
Transmission electron microscopy of tracheary elements (TEs) and fiber cells in *CEP1*-overexpressing (*CEP1*-OE) plants at stage 4. (A) Xylem cells, and high-magnification images of (B) TEs and (C) fiber cells, and the cell walls of (D) TEs and (E) fiber cells. (F) A TE lacking cellular contents and (H) a fiber cell with cellular content. (G) High-magnification image of an empty TE without cellular contents. (I) Vesicles in a fiber cell, showing membrane-like vesicles (solid arrowhead) and tonoplasts (open arrowhead). Scale bars: (A) 10 μm, (B, C) 5 μm, And (D–I) 2 μm. (J) Cell wall thickness of TEs and fiber cells in wild-type (WT) and *CEP1*-OE plants. Sizes of (K) TEs and (L) fiber cells in wild-type and *CEP1*-OE plants. Data are means (±SD) of three replicates.

To investigate the effects of overexpression of CEP1 on xylem development, we evaluated growth and examined cross-sections of *35S*::*CEP1* transgenic Arabidopsis using paraffin sections and TEM. *CEP1*-overexpressing (*CEP1*-OE) plants grew to a similar height as WT plants. The shape of the xylem cells appeared normal and no changes in cell size or wall thickness in TEs and fiber cells were observed in *CEP1*-OE plants compared to the WT (Figs 4, 5). Except for the earliest stage, there were no significant differences in the number of TEs and xylem cells between *CEP1*-OE and WT plants (Fig. 4). The cellular contents had disappeared in the TEs of *CEP1*-OE plants at stage 4 similar to WT plants; however, fewer mitochondria and plastids, as well as membrane-like vesicles, were observed in fiber cells of *CEP1*-OE plants compare to the WT (Fig. 5). These results suggested that overexpression of CEP1 slightly affected PCD in fiber cells but did not alter xylem structure and morphology during stem development.

### Transcriptome data analyses of misregulated genes in the *cep1* mutant

To examine the transcription profile controlled by CEP1 during xylem development, we performed RNA sequencing analyses using mature stems of the *cep1* mutant and WT plants as samples. Overall, 1708 genes were significantly differentially expressed by more than two-fold in the *cep1* mutant (log_2_ ratio ≥−1 or ≤1, FDR ≤0.005), with 733 and 975 down- and up-regulated, respectively, compared to the WT (Supplementary Table S1).

**Fig. 6.**
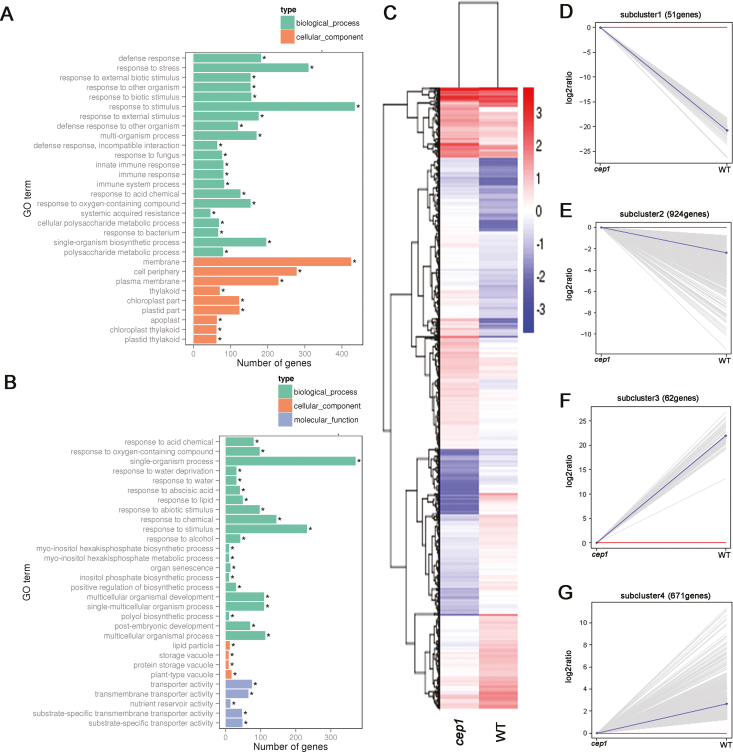
Transcriptome analyses of differentially expressed genes (DEGs). (A) Gene ontology (GO) terms of genes (A) up-regulated and (B) down-regulated in *cep1* mutant plants relative to the wild-type (*P<0.05). (C) Hierarchical cluster analysis of DEGs (*cep1* versus wild-type). Red indicates up-regulation and blue indicates down-regulation. Sub-clusters of down-regulated (D, E) and up-regulated (F, G) genes are shown. Each gray line indicates the relative expression ratio of one gene. Red lines indicate that the gene expression level in the *cep1* mutant is equal to that of the wild-type (WT) (log_2_ ratio=0). Blue lines indicate the mean expression level of all genes in each sub-cluster.

GO analyses revealed that the genes up-regulated in *cep1* were involved in membranes, the cell periphery, and the plasma membrane, which included the biosynthesis of components of the secondary wall such as cellulose, lignin, and xylan, as well as transcription factors and enzymes that regulate the formation of the secondary wall (Fig. 6, Supplementary Table S2). This was consistent with the thicker secondary walls of the *cep1* mutant. Genes encoding proteins stored in vacuoles and involved in cell death were down-regulated in *cep1* (Fig. 6B, Supplementary Table S2), which implied that their misregulation may have been linked to the delayed cellular degradation that occurred during xylem development.

To visualize gene expression patterns in the *cep1* mutant, we performed hierarchical cluster analyses of differentially expressed genes (DEGs, Fig. 6C). The genes were divided into four sub-clusters based on their expression patterns. Sub-clusters 1 and 2 included 62 and 671 genes down-regulated in *cep1*, respectively (Fig. 6D, E), while sub-clusters 3 and 4 included 51 and 924 up-regulated genes, respectively (Fig. 6F, G).

Among the up-regulated transcription factors, the roles of MYB103, MYB63, and MYB46 in regulating secondary wall synthesis have been well characterized, and they were correlated with the thicker secondary walls observed in the *cep1* mutant. Except for *ATHB9/PHV*, the functions in xylem development of the down-regulated genes encoding transcription factors in the *cep1* mutant are not yet well characterized. This group of genes included four homeodomain-leucine zipper (HD-ZIP) family genes (*ATHB7*, *ATHB12*, *ATHB54*, and *ATHB9/PHV*), four *NAM/ATAF/CUC* (*NAC*) family genes (*NAC14*, *NAC16*, *NAC18*, and *NAC29*), and six MYB family genes (*MYB3*, *MYB48*, *MYB59*, *MYB77*, *MYB90*, and *MYB118*) (Supplementary Table S2).

The secondary wall in fiber cells and TEs is composed of cellulose, hemicellulose, and lignin and an increase in any of these components might lead to thicker walls. We therefore analysed the genes involved in their biosynthesis. Cellulose synthase (CESA) proteins are responsible for the synthesis of cellulose and 10 *CESA* genes have been identified in the Arabidopsis genome (Richmond, 2000). Seven CESA genes were differentially expressed in the *cep1* mutant (*CESA1*, *CESA2*, *CESA7/IRX3*, *CESA4/IRX5*, *CESA6*, *CESA7*, and *CESA8/IRX1*), and all of them were up-regulated (Supplementary Table S2) Xylan is the major hemicellulose of the secondary wall in Arabidopsis and glycosyltransferase is involved in xylan biosynthesis (Kumar *et al.*, 2016). Six glycosyltransferase genes that are known to be involved in xylan biosynthesis (Kumar *et al.*, 2016) were up-regulated in the *cep1* mutant, namely *IRX6*, *IRX9*, *IRX10*, *IRX12*, *IRX14*, and *IRX15*. Wall-bound peroxidases and laccases are responsible for the polymerization of monolignols during lignin synthesis (Baucher *et al.*, 2003). Five peroxidases (*PER21*, *PER34*, *PER42*, *PER64*, and *PER71*) and four laccases (*LAC2*, *LAC10*, *LAC12*, and *LAC17*) were up-regulated in the *cep1* mutant, while only one laccase (*LAC15*) was down-regulated.

Microtubules determine the pattern of cellulose by defining the position and orientation of secondary walls during their formation (Wightman and Turner, 2008). Hence, the expression levels of tubulin genes were also analysed, and several of them were up-regulated in the *cep1* mutant, including *TuBA4*, *TuBA5*, *TuBA6*, *TuBB4*, *TuBB5*, *TuBB6*, *TuBB8*, and *TuBB9*, which was consistent with their thicker secondary walls.

Overall, the transcriptional analyses indicated that the up-regulation of genes in multiple pathways of secondary wall synthesis caused the delay in PCD during xylem development and the thicker secondary cell walls in the *cep1* mutant. However, changes to the quantity and structure of individual constituents of the secondary walls in the *cep1* mutant remain to be determined.

## Discussion

The differentiation of TEs and fiber cells involves a cascade of processes including cell wall expansion, secondary wall deposition, lignification, and plant cell death (Turner *et al.*, 2007; Bollhöner *et al.*, 2012). Our results indicated that CEP1 acts as an executor for post-mortem events to clear cell contents during PCD and that it plays an important role in regulating PCD during xylem development. Delayed PCD in *cep1* mutant plants led to an increase in secondary wall thickness in the fiber cells and TEs during xylogenesis (Fig 3).

Premature CEP1 is present in the vacuole in an inactive form, and it is transformed into a mature enzyme in an acidic environment through a proteolytic process of cleavage of pre- and pro-peptides. The mature form plays a role in tapetal degradation and pollen development (Zhang *et al.*, 2014). Activated CEP1 and other hydrolytic enzymes released during vacuolar rupture result in the rapid degradation of cell contents. We speculate that CEP1 has a similar function in PCD during xylem development as tapetal degradation.

The phenotype of the *cep1* mutation suggested that CEP1 is directly involved in xylem PCD. However, because the *cep1* mutation did not completely inhibit differentiation of the TE by PCD, this suggests that CEP1 may not be the only protease involved in xylem PCD. Other proteases may need to catalyse premature CEP1 into its mature active form. It has been shown that γ-vacuolar processing enzyme (γVPE) is critical for maturation of the plant vacuolar protease AtCPY (Rojo *et al.*, 2003). γVPE is a vegetative VPE that is highly expressed in the stem (Gruis *et al.*, 2004), and thus might be an ideal candidate to mediate the maturation process of CEP1 during xylem development. However, whether CEP1 is a direct substrate of γVPE needs to be determined. Alternatively, other proteases may work in parallel with CEP1 to regulate PCD during xylem development. Non-degraded cellular remnants persist in the TE lumen following autolysis in *xcp1* and *xcp1/xcp2* mutants (Funk *et al.*, 2002; Avci *et al.*, 2008). However, whether CEP1 and other proteases are involved in the same pathway or function in independent pathways during xylem PCD remains to be determined.

### CEP1 is involved in co-regulating both PCD and secondary wall deposition during xylem development

Indirect evidence has indicated that pharmacological agents that block xylem cell death also block secondary wall formation (Groover and Jones, 1999; Twumasi *et al.*, 2010). Our data showed that CEP1 not only regulated the degradation of cellular content (Fig. 3) but also affected secondary cell wall deposition during xylem development (Fig. 3S), suggesting that CEP1 co-regulated the processes of cell death and secondary wall deposition. CEP1 acts as an executor in clearing cellular content during PCD, and the digested cellular constituents could be used as substrates for the synthesis of new secondary walls (Groover and Jones, 1999; Helm *et al.*, 2008). Our transcriptome data revealed that many genes related to the biosynthesis of secondary wall components, including cellulose, lignin, and xylan, were up-regulated (Fig. 6); however, these genes may not have been directly regulated by CEP1, but instead may have been to the result of the delayed xylem cell maturation or the thicker secondary cell walls.

We observed more organelles in fiber cells than in TEs in the *cep1* mutant (Fig. 3). This may have been due to differences in PCD and autolysis between the two cell types that have been described previously (Courtois-Moreau *et al.*, 2009; Bollhöner *et al.*, 2012). In TE differentiation, rupture of the vacuole leads to rapid degradation of the cell contents and partial degradation of the primary wall. By contrast, the cytoplasmic contents of fiber cells begin to hydrolyse gradually ahead of vacuolar disintegration, and the cellular debris is retained for a long period of time before it is completely cleared (Courtois-Moreau *et al.*, 2009). Because the duration of PCD in fiber cells is longer than in TEs, this might account for more organelles being observed in in the former during xylem development.

### *CEP1* overexpression slightly accelerates programmed cell death but does not affect secondary cell wall deposition

Fewer organelles were observed in fiber cells and TEs during PCD in the *CEP1*-OE transgenic plants (Fig. 5), suggested that the increased abundance of the CEP1 protein accelerated the PCD process, thus accelerating the deposition of cellular contents on the secondary wall. However, the number and cell wall thickness of xylem cells were not significantly altered in *CEP1*-OE plants compared to the wild-type (Figs 4, 5). Previous research has shown that the speed of degradation of organelles after the rupture of the vacuole during TE differentiation is fast (Obara *et al.*, 2001). Thus, the accelerated PCD process in the TEs and fiber cells was not sufficient to significantly increase the accumulation of cellular contents or to increase the cell wall thickness of xylem cells in the *CEP1*-OE transgenic plants.

Overall, we conclude that CEP1 plays an important role in clearing cellular contents during PCD in xylem development. Mutation of CEP1 results in a delay in PCD and incomplete degradation of the cellular contents. Prolonged PCD provides more material for secondary wall deposition and thus may result in thicker walls during xylem development.

## Supplementary data

Supplementary data are available at *JXB* online.

Table S1. List of genes significantly differentially expressed in the *cep1* mutant compared to wild-type plants.

Table S2. Expression patterns of selected xylogenesis-related genes identified by GO analysis.

Fig. S1. Histological analysis of the basal nodes in the *cep1-2* mutant compared to wild-type plants.

Supplementary Table S1-S2

Supplementary Figure S1
