# Supplementary material for: The papain-like cysteine protease CEP1 is involved in programmed cell death and secondary wall thickening during xylem development in Arabidopsis
Source: J Exp Bot. 2018 Oct 30;70(1):205–15. doi: 10.1093/jxb/ery356 (PMC6305193; doi:10.1093/jxb/ery356)
Supplement: Supplementary Figure S1 [file ery356_suppl_supplementary_figure_s1.pdf]

The papain-like cysteine protease CEP1 is involved in programmed cell death and secondary wall thickening during xylem development in *Arabidopsis*

Running title: The function of CEP1 in xylem development

Jingyi Han[1*], Hui Li[1*], Bin Yin[1*], Yongzhuo Zhang[1*], Yadi Liu[1], Ziyi Cheng[1], Di Liu[1], Hai Lu[1,2**]

1. College of Biological Sciences and Biotechnology, Beijing Forestry University, Beijing, 100083, China

2. Beijing Advanced Innovation Center for Tree Breeding by Molecular Design, Beijing Forestry University, Beijing, 100083, China

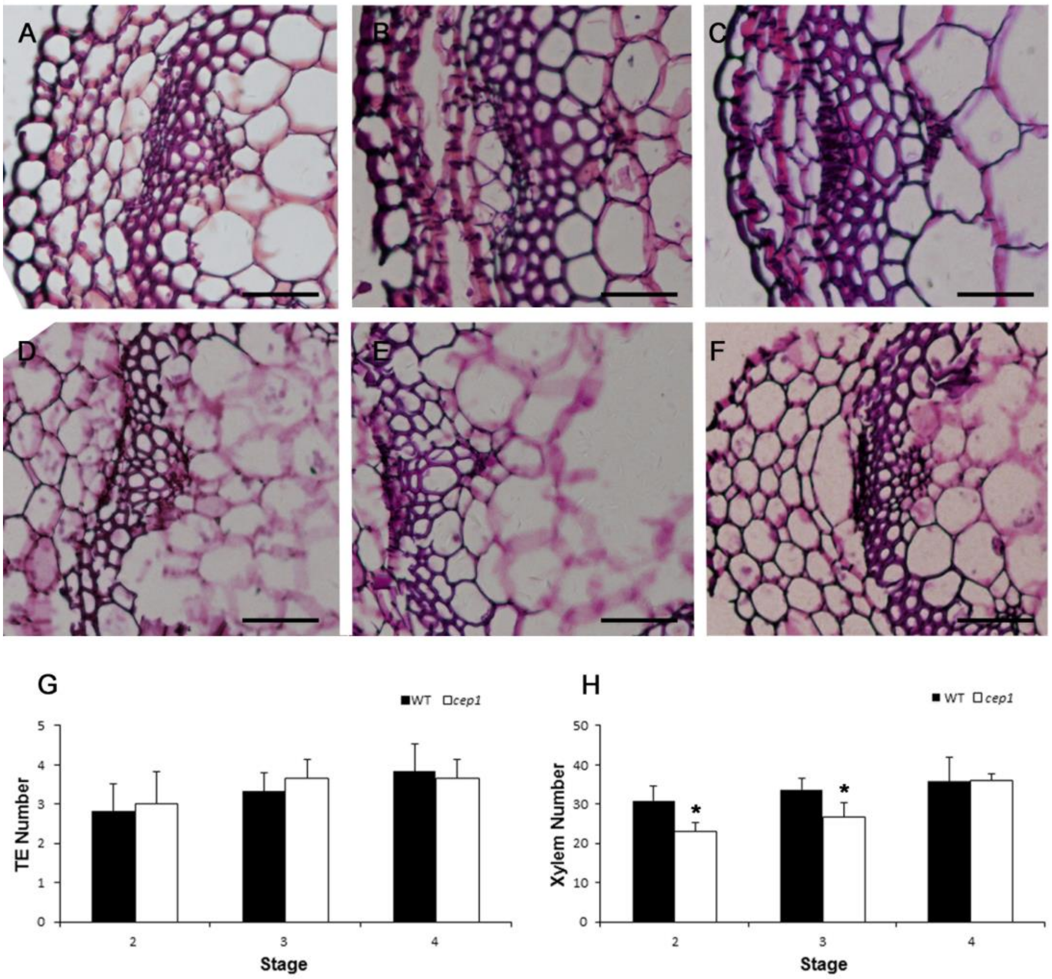

Supplemental Figure 1. Histological analysis of the basal nodes in *cep1-2* mutant

(SALK_137016) compare to wild-type plants was examined. **(A–C)** Cross-section of stem vascular bundles is shown in the wild type from the early to the late stage. Scale bars = 50 μm. **(B–F)** Cross-section of stem vascular bundles is shown in a *cep1-2* mutant plant at stage 2 **(A, D)**, stage 3 (**B, E**), and stage 4 (**C, F**). Scale bars = 50 μm. **(G)** The number of tracheary elements (TEs) in wild-type and *cep1-2* plants. **(H)** The number of total xylem cells in wild-type and *cep1-2* plants.
